# Recognising and responding to acute deterioration in care home residents: a scoping review

**DOI:** 10.1186/s12877-023-04082-y

**Published:** 2023-06-29

**Authors:** Sevim Y. Hodge, Mohammad R. Ali, Ada Hui, Pip Logan, Adam L. Gordon

**Affiliations:** 1 School of Medicine, Centre for Rehabilitation and Ageing Research, University of Nottingham, Nottingham, UK; 2 Department of Cardiovascular Sciences, University of Leicester, Leicester, UK; 3 School of Health Sciences, University of Nottingham, Nottingham, UK; 4 Division of Medical Sciences and Graduate Entry Medicine, University of Nottingham, Nottingham, UK

**Keywords:** Care homes, Acute deterioration, Residents, Recognition & response, Managing acute care in residents

## Abstract

**Background:**

Acute deterioration describes a rapid change in physical and/or mental health resulting from an acute illness – e.g., heart attack or infection. Older people in care homes are some of the frailest and vulnerable in society. They have complex health needs, experience multiple long-term conditions (MLTC) and have weakened immune systems due to the ageing process. They are more susceptible to acute deterioration and delayed recognition and response, is linked to poorer health outcomes, adverse events and death. Over the past five years, the need to manage acute deterioration in care homes and prevent hospital admissions has led to development and implementation of improvement projects, including the use of hospital derived practices and tools to identify and manage this condition. This is potentially problematic as care homes are different from hospitals—options to escalate care vary throughout the UK. Further, hospital tools have not been validated for use in care homes and have shown to be less sensitive in older adults living with frailty.

**Objectives:**

To collate the available evidence on how care home workers recognise and respond to acute deterioration in residents using published primary research, non-indexed and grey literature, policies, guidelines and protocols.

**Methods:**

A systematic scoping review was conducted following Joanna Briggs Institute (JBI) scoping review methodology. Searches were conducted using: CINAHL (EBSCOhost), EMCARE (OVID), MEDLINE (OVID) and HMIC (OVID). Snowball searches of included studies’ reference lists were conducted. Studies that featured care homes with or without nursing and provided 24/7 care to residents were included.

**Results:**

Three hundred and ninety-nine studies were identified. After reviewing all studies against inclusion criteria, *n* = 11 were included in the review. All studies used qualitative methods and were conducted in Australia, UK, South Korea, USA and Singapore. Four themes were generated from the review: identifying residents with acute deterioration; managing acute deterioration, care home policies and procedures, and factors affecting recognition and response to acute deterioration.

**Findings:**

Recognition and response to acute deterioration in residents is determined by multiple factors and is context sensitive. There are several interrelated factors within and external to the care home that contribute to how acute deterioration is recognised and managed.

**Conclusions and Implications:**

The available literature on how care home workers recognise and respond to acute deterioration is limited and often subtends other areas of interest. Recognising and responding to acute deterioration in care home residents is reliant on a complex and open system encompassing multiple interrelated components. The phenomenon of acute deterioration remains underexplored and further research is required to examine contextual factors that accompany identification and management of this condition in care home residents.

**Supplementary Information:**

The online version contains supplementary material available at 10.1186/s12877-023-04082-y.

## Background

The global population is ageing. An estimated 2 billion people will be 65 or older, and 400 million 80 or older, by 2050 [1]. An anticipated consequence is an increase in the number of older adults living with multiple long-term conditions (MLTCs), age-related diseases and frailty [2]. This will lead to sustained and increased demand for care services, including care homes [3].

Care homes are long-term care facilities, with or without nursing support, which are homes to people requiring 24-h care to support activities of daily living. Most residents live with MLTCs and frailty, many live with dementia, cancer, stroke and heart disease [4]. Consequently, care home residents are at high risk of experiencing acute deterioration [5, 6]. Acute deterioration describes a sudden decompensation of physiological and/or mental status [7]. It can result from acute illness, or exacerbation of a chronic condition. It is potentially avoidable and treatable if recognised and responded to promptly [6, 8–10].

Signs and symptoms of acute deterioration may include, but are not limited to, changes in respiratory rate, oxygen saturation, heart rate or rhythm, blood pressure, mental state, skin perfusion, urine output, or temperature [6, 8, 11]. In hospital, early warning score (EWS) assessment tools have been developed and are widely used to identify patients with acute deterioration [12, 13]. These use physiological observations and aggregate scoring systems; with a higher score indicating greater clinical risk of adverse outcomes including injury and death [12]. Systematic implementation of early warning scores has been associated with reduced mortality in hospital and pre-hospital settings [14, 15].

More recently, improvement projects have incorporated EWS systems into care homes [16, 17]. However, it is unclear whether these assessment tools work as well in these settings. The physiological frailty, disability and cognitive impairment of care home residents may mean they manifest acute deterioration in different ways, whilst differing skills and competencies of care home staff may influence the sensitivity and specificity of such approaches [18]. Additionally, changes to care home processes and routines as a result of implementing hospital approaches may have unintended adverse consequences, including depersonalisation of the care setting and opportunity cost for staff who are faced with the choice of prioritising observations or person-centred care [19].

In preparation for work to develop care home specific ways of recognising and responding to deterioration, we set out to identify research, policy, guidelines and protocol papers outlining how care home staff recognise and respond to acute deterioration in residents.

## Objectives

To identify published primary research and grey literature including policies, guidelines, and protocols regarding how care home staff:

i. identify an acutely deteriorating resident
ii. respond to an acutely deteriorating resident
iii. use protocols or tools to help deliver care to an acutely deteriorating resident

## Method

We conducted a systematic scoping review in accordance with Joanna Briggs Institute (JBI) methodology and used the PRISMA-SCR reporting checklist [20]. The review was registered with JBI Evidence Synthesis, and the full protocol published [21].

Preliminary searches were conducted in January 2019 to identify any existing reviews and locate any relevant clinical guidelines or protocols relating to acute deterioration in care homes. The following electronic platforms were searched: PROSPERO, the Cochrane Library JBI evidence-based practice database, Google Scholar, CINAHL and MEDLINE using the terms “deterioration” and “care homes”. This search yielded few responses in relation to acute deterioration in care homes. No current scoping or systematic reviews were identified. However, two systematic reviews were identified [11, 22] which considered care home deterioration from context of acute secondary care. Additionally, care home stakeholders who were part of the study steering group were asked to recommend any care home specific resources used to support the management of acute deterioration in residents. Stakeholders suggested the Gold Standard Framework [23] and the Enhanced Health in Care Homes [24, 25]. These were excluded as they are resources to manage the care of older people entering the end stages of their lives/were not specific to acute deterioration in care homes.

### Search strategy

The search strategy was developed with an information scientist to ensure index and key terms were captured and that searches were conducted in consistent ways across multiple databases [26]. The following electronic databases were searched: CINAHL (EBSCOhost), EMCARE (OVID), MEDLINE (OVID), and HMIC (OVID). To reflect the reconfiguration of healthcare services to explicitly focus on acute deterioration following the Royal College of Physicians’ launch of the National Early Warning Score (NEWS) [13] and our published protocol [21], a time filter was applied to database searches from January 2009 – February 2023. Snowball searches were conducted using the reference lists of included studies.

We used the following search terms: [‘nursing home’ OR ‘residential home’ OR ‘care facility’ OR ‘residential facilities’ OR ‘assisted living facilities’ OR ‘homes for the aged’ OR ‘residential aged care facility’] AND [‘clinical deterioration’ OR ‘rapid’ OR ‘acute’ OR ‘unwell’ OR ‘deterioration’ OR ‘recognise’ OR ‘identify’ OR ‘response’]. Medical Subject Headings (MeSH) terms were used where available. The full search strategy for MEDLINE can be viewed in Additional file 2: appendix II. The final searches were completed on 22 February 2023.

### Data extraction & analysis

The title and abstract of each record were reviewed by two researchers (SH and MA) using the eligibility criteria outlined in Table 1. Data were extracted by the primary author (SH) using a modified JBI data extraction tool (Additional file 1: appendix I) and checked by the second reviewer (MA) for consistency. Study characteristics, population, concept, context and main findings in relation to review objectives were extracted from included articles. Snowball searches of included studies’ reference lists were conducted. Included studies were read in full a minimum of three times. Data were analysed thematically using a hybrid of deductive and inductive coding [27, 28] to identify patterns/themes. The approach consisted of using the review questions as an initial, a priori framework to review the data. The researchers also remained open to patterns within the data pertinent to identifying and managing acute deterioration that fell outside the scope of the review questions. All studies were coded manually by author SH and a log of codes generated. Similar codes were grouped together into subthemes and further grouped into main themes. To ensure methodological rigour in analysis, codes and themes were reviewed by author MA and reflexive discussions took place between researchers.

**Table 1:** Study eligibility criteria

	**Eligibility criteria**
**Population**	Directly related to care homes/care home staff responsible for providing direct care to residents e.g. RNs, AHPs, unregistered staff employed by care home. The review focused on care homes specialising in caring for older adults aged ≥ 65 years
**Concept**	Records featuring how care home staff recognised/responded to acute deterioration from the perspectives of care home organisations and staff
**Context**	For the purposes of the scoping review ‘care homes’ included facilities that provide long-term care to residents. This included care homes with/without nursing care. Temporary or respite care home facilities were excluded
**Types & sources of evidence**	Primary research, grey/non-indexed sources, frameworks, assessment tools, policies and guidelines. Sources that reported on implementation projects, interventions or outcomes relating to care homes but did not specifically engage or capture how care home workers recognised or responded to acute deterioration were excluded Opinion pieces, editorials, conferences, stories, abstracts, and editorials were also excluded

## Results

**Fig. 1:**
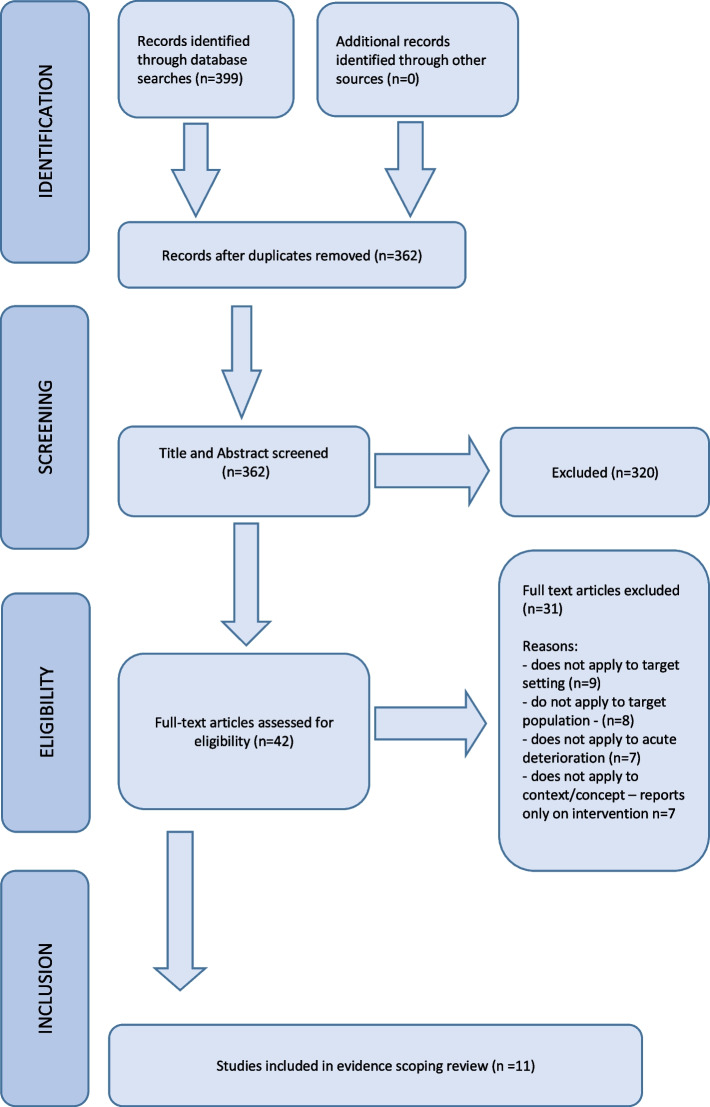
PRISMA flow chart of study search and selection process

The search yielded 399 articles after deduplication. After title and abstract screening, 362 were excluded. The full texts of 42 studies were reviewed and a further 33 were excluded at this stage. A meta-synthesis [29] regarding decisions to transfer residents to hospital was screened. This was excluded to avoid duplicating citations as the relevant sourced articles (*n* = 2) were included. A total of *n* = 11 eligible studies were included. No relevant national policy or guidelines were identified from the searches. A PRISMA diagram is shown in Fig. 1.

Characteristics and key findings of included studies are summarised in Table 2. All were qualitative studies except for one mixed-methods study that utilised quantitative and qualitative approaches [17]. They comprised: semi-structured interviews plus observations [30–32]; semi-structured interviews [17, 33–36]; individual interviews/focus groups [37] and focus groups [38, 39] with care home staff. Five studies focused on decision-making surrounding transferring residents to hospital [32, 34–37]. Four studies captured clinical decision making/management of acutely unwell residents [30, 31, 33, 39]. One study used qualitative interviews to examine how resident deterioration is managed following introduction of a hospital avoidance programme [38]. One mixed-method study evaluated the introduction of NEWS in care homes – only the qualitative data have been included in this review as only these data met the eligibility criteria of the study [17]. Studies were conducted in Australia *n* = 5; UK *n* = 3; USA *n* = 1; South Korea *n* = 1 and Singapore *n* = 1.

**Table 2:** Charted data of study characteristics and key findings

**Author/year/country/title**	**Methods**	**Sample size**	**Key measures**	**Substantial findings**
**Lopez, R. (2009) **[30] **USA** **Decision making for acutely ill nursing home residents: nurses in the middle**	Grounded theory Semi-structured interviews with NH staff 74 h of observations in relation to Nurses’ responses to acute deterioration Data analysis plan: constant comparison analysis, iterations to interview questions informed by data to reflect/test data generated hypotheses	Nurses from four NHs based in suburbs Total: *n* = 10 RN = 6 LPN = 4	Grounded theory to explore and describe decision-making process of NH staff when managing acutely unwell residents and develop a theory grounded in these experiences To develop a theory to that that explains social psychological issues and processes	The social problem experienced by nurses was how to create a plan for residents’ with acute illness whilst that was acceptable to all stakeholders involved e.g. doctors, family, and the resident The social psychological process in order to manage the problem was identified as nurses trying to satisfy all parties. This had four phases – weighing the significance, notifying the family, feeling it out and playing the middleman Residents’ resuscitation status and family wishes were significant in deciding active or palliative care than the acute event
**Shanley, C., Whitmore, E., Conforti, D., Masso, J., Jayasinghe, S. and Griffiths, R. (2011) **[36] **Australia** **Decisions about transferring nursing home residents to hospital: highlighting the roles of advance care planning and support from local hospital community health services**	Semi-structured interviews Data analysis plan: content related coding based on interview schedule. Then thematic analysis	Nursing Home Managers *n* = 41	To explore and identify current practice and improvement opportunities regarding decisions to transfer residents to hospital	How acute deterioration is identified is not mentioned Transfer to hospital is both part of managing acute deterioration and an outcome. The study lists in-house protocols for when residents with acute illnesses may be transferred to hospital. Such as shortness of breath or chest pain. No quotes provided to support this claim Lack of resources such as adequate staffing or external healthcare support was reported as a reason for hospital admission. It is unclear whether this is directly related to acute deterioration illnesses or all aspects of a resident’s health
**O'Neill, B., Dwyer, T., Reid-Searl, K. and Parkinson L. (2016) **[39] **Australia** **The deteriorating resident in residential aged care: A focus group study**	Focus groups × 4 with nursing staff from residential aged care facility (RACF) Data analysis plan: inductive thematic analysis	Staff from 94 bed RACF Total *n* = 49 PC *n* = 30 EN *n* = 7 RN *n* = 12	To explore nursing staff’s perceptions of managing deteriorating residents to support future developments of hospital avoidance programmes	Knowing residents well supported early detection of acute deterioration. Especially with PC’s who cared for residents daily and had frequent interactions with them Part of managing condition was escalating care. There was a system within the care home associated with staff role and responsibility and a system for communicating with external healthcare providers. Nurses are at the centre of these conversations Workload implications – cannot manage routine care of residents whilst managing acutely unwell residents Lack of equipment and resources was felt to contribute to delays in identifying/managing acute deterioration Value of nursing assessment not recognised by residents, relatives and external health professionals
**O'Neill, B., Dwyer, T., Reid-Searl, K. and Parkinson L. (2017) **[38] **Australia** **Managing the deteriorating nursing home resident after the introduction of a hospital avoidance programme: a nursing perspective**	Focus groups × 3 Purposive sampling – clinical staff involved directly with in delivering nursing care to residents Data analysis plan: thematic analysis of focus group data	Total *n* = 21 RN/EN *n* = 8 NA *n* = 13 RN/EN roles not defined. Both regulated nurses in Australia but distinction between RN/EN role not given RN/EN grouped and referred to as nurses in focus groups	Aim: Examine how deterioration is managed following the introduction of a hospital avoidance programme Focus group examining staff perceptions regarding their management of the deteriorating resident Conducted 14–15 months post implementation Focus group participant ranged from 5–10. Duration: 30-45 min Focus group 3 – only NA participants in this group. They were only asked questions in relation to evaluating the hospital avoidance programme Context: Introduction of a programme to identify and manage acute deterioration. Included diagnoses and clinical indicators of AD, use of supportive equipment Inductive thematic analysis – 30 codes identified, grouped under 6 main headings then refined to 5 main themes Findings discussed with research team and shared in a final 40-min focus group with 5 participants to increase credibility and ensure representativeness of findings	The intervention provided structure for NH staff as equipment and resources was provided to identify, manage and monitor deterioration The programme appeared to increase staff’s confidence in their decision making Workload and other organisational factors hindered detection and management of deterioration and had subsequent effects on the care of other residents e.g. staffing Staff felt more confident and integrated in residents' care delivery post intervention
**Laging, B., Kenny, A., Bauer, M. and Nay, R. (2018) **[31] **Australia** **Recognition and assessment of resident deterioration in the nursing home setting: A critical ethnography**	Ethnography 184 h observations—66 participants 10 General Practitioners 10 Registered Nurses 10 Enrolled Nurses 8 Personal Care Assistants 5 residents 5 relatives qualitative interviews – 40 study August 2012-May 2015	× 2 RACF NH1—30 beds 1 RN: 30 residents NH2—75 beds 1RN: 75 residents *n* = 66 observations *n* = 40 interviews	Observations during all aspects of care except during personal hygiene or toileting Qualitative interviews × 40 developed from observation data	RNs were reliant on PCA/ENs to escalate concerns to them. This was due to very high resident to nurse ratio Organisational structures impacted care delivery and contact with residents. Familiarity and continuity of care with residents enabled staff to notice a change in a resident, however, daily routines hindered this process Registered nurses rarely completed physical observations at a time when a resident was found unwell. They lacked confidence in their ability to clinically assess residents. They followed care home policies strictly – this was driven by fear of litigation but did little to support the nurse's autonomy
**Amadoru, S., Rayber, J., Rajni, J. and Yates, P. (2018) **[37] **Australia** **Factors influencing decision-making processes for unwell residents in residential aged cate: Hospital transfer or Residential inReach referral (RiR)?**	AIM: To investigate decision-making around hospital transfer and/or referral to RiR RiR is a geriatrician led service. 7 days per week – 9am-5 pm. Includes advice, sub-acute interventions (IV antibiotics, fluid resuscitation, acute medical admissions, palliation, and other clinical care) Semi structured Individual and group interviews with residential aged care facilities staff (RACF), general practitioners (GPs) and registered nurses (RN)	*n* = 40 interviews *n* = 31 from RACF staff *n* = 5 GPs *n* = 4 RiR RNs RACF’s with High, middle, low referrals to ED RACFs *n* = 8 Not-for-profit *n* = 2 For profit *n* = 6 *n* = 6 RACF > 80 beds *n* = 2 RACF 60–80 beds	Perspectives of RACF staff, GPs providing medical management in RACFs and RiR RNs Decision-making around hospital transfer or referral to Resident in-Reach (RiR) programme in acute deterioration residents Factors affecting decision-making when residents become unwell The roles of RiR services and locum GP care Hospitalisation in managing residents Strengths & weaknesses of RiR service	RiR – resource for RACF can use to manage care of acutely unwell residents. Reasons for using the services was for assistance with caring for residents with sudden clinical deterioration. However, RACF staff reported that some conditions fell outside of the scope of RACF e.g. haemorrhaging Participants liked having a team that could assess an unwell resident and provide sub-acute/acute care. Their decision to refer to this team formed part of their assessment and management criteria How unwell residents are identified is not explored in detail Several factors were considered before care was escalated to the RiR/or how they responded—included RACF policies or systems, cannot access resident's GP, complications with ACPs and family attitudes Decisions around the management of deterioration are complex as involved several components such as: resident and family requests, Complexity of care, variability in care (includes access to equipment and assessment tools)
**Hodgson, P., Greaves. J., Cook, G., Fraser, A. and Bainbridge L. (2021)** **UK** **A study to introduce National Early Warning Scores (NEWS) in care homes: influence on decision making and referral processes**	Mixed-methods study – convergent parallel design Quantitative data – comparisons between NEWS scores, activities of daily living and frailty index scores Qualitative interviews with care home staff 6-weeks after implementation and training for the use of NEWS in care home Data analysis plan: Quantitative—descriptive statistics followed by correlation outcomes using chi-square calculations Qualitative – Thematic analysis	Participants from *n* = 4 care homes Quantitative – *n* = 276 residents Qualitative – Total *n* = 13 Manager *n* = 1 Deputy Manager *n* = 1 Nurse n = 1 Senior care assistant *n* = 5 Care assistant *n* = 5	Evaluate impact of NEWS scores alongside frailty and activity of daily living indexes Investigate care home staffs’ experiences of using NEWS in care homes	Concept of acute deterioration is captured in relation to use of NEWS. How acute deterioration is identified is not explicitly discussed Prior to implementation of NEWS staff identified acute symptoms through subjective observations. Does not confirm if NEWS if more efficient/able to identify acute deterioration Despite having a tool to help identify and manage acute deterioration, staff still relied on ‘knowing the resident’ as the main indicator for them being unwell NEWS cannot act as a diagnostic tool in care homes due to the complex nature of care home residents. However, using the tool was reported as empowering staff and was a useful aid when communicating their concerns
**Choi, Y-R and Chang, S. (2022) **[33] **South Korea** **Nurses’ conceptualizations of managing emergencies in nursing homes**	Qualitative interviews with nurses from five different nursing homes Data analysis plan: phenomenography – identification of categories followed by the development of logical relationships	*N* = 20 Nurses from 5 care homes Minimum 2 years’ experience working in care home	To describe and identify nurses’ perceptions of their management experiences in nursing home emergencies	Phenomenography used to describe the different ways in which individuals experience to the phenomenon of interest Managing acute deterioration was dichotomised as responses in an emergency scenario and daily routines to prevent further deterioration of a deterioration from happening Emergencies were defined as scenarios that threatened life of the resident and requires immediate treatment Nurses described any physical changes, expressions or behaviours in a resident were indicative of a potential emergency and prompted a series of assessments. These variations were considered different from ‘typical’ days Nurses assessment of residents in a potentially life threatening scenario was based on the residents symptoms at the time and comparing this with their medical history/long term conditions Prevention of acute deterioration was based on residents’ medical history, daily checks and in some cases changes in their care plans. For example, increased observations and trying to maintain observations (vital signs) within normal parameters
**Nguyen, P., Liaw, S., Tan, A., Bin Rusli, K., Tan, L., Goh, H. and Chua, W. (2022) **[34] **Singapore** **Nurses caught in the middle: a qualitative study of nurses’ perspectives on the decision to transfer deteriorating nursing home residents to emergency departments in Singapore**	Individual semi-structured interviews with nurses from one nursing home Data analysis plan: thematic analysis	Total *n* = 14 RN *n* = 9 EN *n* = 2 Nurse manager *n* = 3	To explore nurses’ experiences surrounding transferring acutely unwell residents to the emergency department	Staff knowledge of their residents was key to them identifying changes in a resident’s health status. Knowing their normal behaviours/characteristics was a reference point to spotting changes but was also indicative of a potential emergency situation Geriatric knowledge and applying this to their residents supported skilled assessment of residents. Part of the management was transferring residents to hospital when they could not be managed in the home Specified conditions warranted immediate transfer to hospital e.g. oxygen desaturation Nurses described as an intermediary when managing acutely unwell residents and family wishes or involvement influenced care and transfer decisions
**Harrad-Hyde. F., Armstrong, N. and Williams, C. (2022) **[35] **UK** **Using advance and emergency care plans during transfer decisions: a grounded theory interview study with care home staff**	Semi-structured interviews with care home staff across six care homes Data analysis plan: Straussian approach to grounded theory	Care homes *n* = 6 NH *n* = 3 RH *n* = 2 Dual registered *n* = 1 Total *n* = 30 Manager *n* = 7 Deputy manager *n* = 3 RN n = 7 Senior carer *n* = 7 Carer *n* = 6	To describe care home staff’s experiences and perceptions of using written plans during in-the-moment decision making about potential resident hospital transfers	Staff preferred to keep residents in the home but feared litigation Family involvement influenced decisions to transfer residents to hospital ACP/DNAR status – used as a means or preparing for a deterioration. Although did not cover all scenarios involving acute deterioration. There were challenges around an acute infection and family members wanting the resident to have active treatment despite having an advance care plan and do not resuscitate order Written plans helped legitimise decisions to transfer or not transfer residents to hospital
**Harrad-Hyde. F., Armstrong, N. and Williams, C. (2022)** **UK **[32] **‘Weighing up risks’: a model of care home staff decision-making about potential resident hospital transfers**	Semi-structured interviews with staff from six care homes Ethnographic observations Data analysis plan: grounded theory to develop a model of the decision-making process	Interviews *n* = 30 Observations *n* = 113 h	To develop a conceptual model to describe care home staff’s decision-making when faced with a resident who potentially requires hospital transfer	Hospital transfers were conceptualised as the outcome to a process that occurred in the care home How acute deterioration is identified is not discussed. Knowing the resident is described as helping to spot changes and raise any concerns However, the management and decision to transfer the resident to hospital, ask for help from GP or call from an ambulance was the decision of the RN Layers of hierarchy present in all homes. There was an internal hierarchy that each staff member followed when raising concerns about an unwell resident Hospital transfer followed after a series of escalations – as last resort or only manageable option Specific conditions were ‘required immediate transfer to hospital e.g. head injuries and fractures. These were protocolised formally and informally (governed by care home norms)

## Thematic analysis results

Four main themes, comprising 34 codes (Table 3), were identified: identifying residents with acute deterioration; managing acute deterioration; care home policies and procedures; and factors affecting recognition and response to acute deterioration.

**Table 3:** Review questions, codes and themes

**Review question**	**Deductive analysis**	**Associated codes**	**Theme topic**	**Example quote**
How do care home staff recognise acute deterioration?	How do care home staff recognise acute deterioration?	- Recognise/see (change in baseline norm) - Identified a new problem/illness - Gut feeling – suspect something is wrong - Knowing the resident - Norm/usual resident	1. Identifying residents with acute deterioration	*“We work with the residents every day. We know their routines; we know their characters and we know when there is something wrong…” H–H B p5* *“Normally we can see if a resident is okay because we care for them every day…” Nguyen p.433* *“PCAs will pass general comments – “room number 53, she looks pale”. They mention it in passing without realising this could be something happening” (Amadoru: p.1456).* *“We know our residents, if you’re going with your gut – go with your gut. That doesn’t change” (Hodgson p.524) *
How do care home staff respond to acute deterioration?	How do care home staff respond? What happens? What actions are taken?	- Respond—actions taken – (deliver care task e.g. medication, observations, assessment) - Inaction – ignored/not escalated - Watched/reassess - Escalation of care - Reporting of incident/condition - Following in-house protocol - Ask for help - Hierarchy - Hospital transfer	2. Managing acute deterioration	*“I see this dressing full of blood and smelling. I said to the PCA – didn’t you see this? And the other PCA said “Yes, but what am I supposed to do about it? “Yes, but what am I supposed to do about it? That’s the nurse’s job…”” (Amadoru: p.1457).* *“…I see the workload is not going to be manageable. So I have sent a couple [of residents] to hospital” (Shanley et al.,2011 p.2901).* *“That day, we noticed her left side couldn’t move like before and we referred her to ED suspecting it was a stroke” (Nguyen et al., 2021 p.433).* *“Statements from a few nursing assistants who felt they were now working more closely with the nurses as a resident’s health was being monitored…one nursing assistant described her role as being more integrated into the process” (O’Neill, 2017: p.317).* *“…they are confident in us that we’ve done [assessed the resident using NEWS] properly” (Hodgson et al., 2021 p.523).*
What protocols/tools do care home staff use to help deliver care to an acutely deteriorating resident?	Is there evidence of protocols or in-house procedures used to help identify and manage acute deterioration? How are they used? How to they help?	- In-house protocol/policy, - Assessment tools, - Guidelines - Care home directed - Routine care	3. Absence of acute deterioration protocol and tools	*“I think protocols, for some nurses, it just becomes a process of ‘don’t think, just do” (Laging et al.,2018 p.1458)* *“Because a very small percentage can go pear-shaped [registered nurses] are now taught make sure you refer on…and not using their clinical acumen to the same degree” (Laging et al., 2018 p.1458)*
	**Inductive analysis**			
	Other interrelated patterns that were from the data.	- Size (number of beds), - Staffing ratios - Access to external help and resources - Care home culture - Fear of litigation	4. Factors affecting recognition and response to acute deterioration	*“It’s hard sometimes, you’re trying to really hold out for the inReach [RiR] service. You can’t get hold of the GP and you get a locum in the middle of the night saying, ‘send to hospital’” (O’Neill, 2017: p.65).* *“The [transfer I can think of, was where there was a little break in communication. The care plan didn’t actually state ‘not for hospital admission’” H-H* *“We can’t not [transfer], because it’s too litigious not to transfer them…” (Shanley et al., 2011 p.2901).* *“ACP…is not a legal document…it’s just guidelines for you when emergency situations arise” (Nguyen et al., 2021 p.434).*

### Identifying residents with acute deterioration

Reports on how care home staff recognised acute deterioration in residents varied. Central to this theme were descriptions of care home staff relying more on instinct rather than structured tools or guidelines to recognise acute deterioration. Six studies reported ‘knowing the residents’ and their ‘baseline norms’ as enabling them to identify when a resident became unwell [17, 32–34, 36, 39]. This was often reported in conjunction with care home staff describing ‘intuition’, a ‘gut feeling’ or ‘just knowing’ when a resident’s health status had deviated from their norm [32, 39]. Furthermore, identification of acute deterioration was reported as care home staff relying on tacit knowledge and their relationship with residents as opposed to conscious logical rationale [31, 34, 39].

O’Neill et al.’s., (2017) study examining nursing home staff’s perceptions of managing acute deterioration following the introduction of a ‘sub-acute program’ reported on a hospital style approach to identifying and managing acute deterioration [38]. The sub-acute program included training on signs and symptoms of acute deterioration, associated illnesses/diagnoses, and use of equipment to manage acute deterioration in residents. Even with this additional knowledge and training, participants still reported a ‘sense of knowing’ when a resident was unwell. Explicit examples of how acute deterioration was identified following the implementation of the sub-acute program were not provided and it remains unclear how/if the intervention improved identification of residents with acute deterioration or if care home staff relied on their knowledge of the resident to detect changes in health. The study also found that detection of acute deterioration was increased because of raised awareness of signs and symptoms and increased confidence in clinical intuition. However, staff reported being competent at recognising when a resident was unwell prior to the programme being implemented. The assertion that the subacute programme increased recognition is not grounded in evidence and it unclear how the authors knew this or how the fact staff were “good” was objectively established [38]. Similar findings were reported in Hodgson et al.’s., (2021) study evaluating the introduction of NEWS in care homes. Staff were interviewed from two care homes, and they reported knowing their residents remained the ‘main source’ of identifying a possible acute deterioration rather than using NEWS [17].

Studies collectively reported reliance on unregistered care staff to identify acute deterioration [31, 32, 36, 37, 39]. Registered Nurses (RNs) were reported as having limited interactions with residents, with most day-to-day contact from Enrolled Nurses (ENs) and Personal Care Assistants (PCAs). RNs had less opportunity to identify acute deterioration because of limited resident contact and the need to work through intermediaries with varying skills and experience. Four studies reported RNs concerns about relying on unregistered staff to identify acute deterioration as early clinical indicators might be missed due to PCAs’ lack of clinical acumen or training [31, 36, 38, 39].

Overall, these concerns reflected a lack of confidence amongst RNs about delegating and/or relying on unregistered staff to identify acutely unwell residents. Countervailing against this was evidence about the importance of PCAs and how they are nurses’ ‘eyes and ears’, best placed to notice changes in residents [31].

### Managing acute deterioration

Central to this theme were the common listed actions/series of events undertaken by care home staff to manage acute deterioration and the support available from external healthcare providers (Table 4).

**Table 4:** Responses to acute deterioration reported in included studies

**STEP 1 – response to AD initiated by care home staff**	**STEP 2 – response to AD by external healthcare visitor**
Personal care assistants/enrolled nurses reporting concerns to RN	Care home staff advised to call for ambulance services (out of hours)
Clinical observations taken – vital signs (BP, HR, RR, O2 etc.)	Direct call to ambulance services
Request/referral for GP review	Decision to transfer resident to hospital
Call for ambulance services	Review/follow ACP directive
Decision to transfer resident to hospital (out of hours)	Prescribe/administer sub-acute care (antibiotics, IV fluids)
Follow in-house care home policy	Decision to initiate palliative treatments
Follow ACP directive	GP review of unwell resident
Request for RiR team review	
Discussion/concerns raised regarding palliative care	
Monitor resident – watch & wait	
In-action – concerns not escalated	
Acknowledgement of resident/relative requests	
Use of SBAR communication tool to escalate concerns	
Response to AD influenced through fear of litigation	

Five studies reported on resident transfer to hospital as part of the management plan of a resident but also as an outcome of acute deterioration [32, 34–37]. Care home staff were reported as aspiring to keep residents in their home because hospitals were considered ‘traumatic’ for residents and staff worried they would not receive high quality and holistic care. Hospital transfers were described as a ‘last resort’ which happened due to lack of adequate resources to manage the condition in the home, emergency scenarios that ‘threatened life’, or family/resident requests. The same issues were reported in studies that had additional services in place to help manage acute deterioration and avoid hospital transfer [17, 37, 38]. O’Neill et al., (2017) found their sub-acute program provided structure for care home staff to assess and provide care for residents in situ in the event of deterioration [38]. Participants stated that their responsibilities had increased however, their time was used differently – focus was directed to providing care to a resident that had deteriorated instead of organising a hospital transfer. Whether the sub-acute programme made it more likely that staff would ask for help and escalate care or, if it prevented hospital admissions was unreported.

Amadoru et al., (2018) reported care homes having access to a purpose-designed ‘rapid-in-reach service’ that could provide assistance for residents with acute deterioration [37]. The RiR service comprised nurse specialists and geriatricians and provided ‘sub-acute care’ to residents in the care home. The study reported RNs/ENs frequent use of the services, especially when access to usual GPs or other health professionals was limited. Not only did this help care home staff, the RiR reduced GP workload, and helped residents who might not be able to access GPs during busy times when they had competing clinical priorities. Outside of the RiR service hours, the study reported problems with accessing timely care for residents and this increased likelihood of residents being sent to hospital.

Registered nurses were considered the highest-ranking figure when managing a resident with acute deterioration within the care home. RNs were described as having overall responsibility and decision-making authority in these scenarios despite having infrequent interactions with residents [31–33, 35]. A recurrent pattern identified was RN and PCA role boundaries and how this interfered with managing acute deterioration. On some occasions, this was considered to have a negative impact on resident care and potentially contributed to delayed responses to acutely deteriorating residents [31, 37].

Five studies demonstrated a shift in hierarchy when care home workers requested services from external healthcare providers [31, 32, 35, 37]. The ‘handing over’ of authority and decision making from RNs/ENs/PCAs to attending external healthcare professionals was evident in all studies. This passing of responsibility was not explicit or even conscious. Staff appeared to assume this position when they lacked confidence to lead/manage care of the residents, had litigation concerns, experienced pressure from residents or relatives, and external healthcare professionals undervaluing care home staff’s assessments which was evident in all studies. An exception to this was Hodgson et al., (2021) study which reported the use of NEWS empowered and validated care home staff’s assessments as all involved parties were speaking the same language [17].

## Care home protocol and procedures

Scant evidence demonstrated the use or existence of acute deterioration protocols in care homes [17, 30–39]. However, conditions such as haemorrhage, head injuries, fractures, respiratory distress and falls were protocolised as needing immediate transfer to hospital [31, 34, 36, 37, 39]. Furthermore, care home staff reported occasions where they felt obligated to send residents to hospital regardless of whether this was deemed clinically appropriate. The main reasons for this were described as fear of litigation/disciplinary action, perceived lack of confidence in the ability to perform clinical assessments or as a way of managing other care home pressures such as continuing routine care alongside the demands associated with an acutely unwell resident [34, 36, 37, 39]. A consequence was reported as the potential loss of clinical autonomy and a deskilled of a workforce unable to conduct assessments of unwell residents [31].

### Factors affecting recognition and response to acute deterioration

Central to this theme were multiple interrelated factors influencing identification, escalation and management of acutely unwell residents e.g. organisational factors (staffing ratios and skill mix), access to training and equipment, and care home culture (Table 5). These factors were not part of the initial review objectives however, they were prominent in all studies and provided essential context to understanding the management of acute deterioration in care home residents.

**Table 5:** Factors influencing the care of residents with AD

**Organisational factors (care home directives/policies)**
Polices/directives (includes major health conditions, litigation, performance management)
Advanced care planning (ACP) – record of residents’ treatment and care wishes
Resident/family requests – influenced care of resident with AD. Overruled ACP/clinical assessments, enables requests for hospitalisation
Staffing levels and skill mix – care homes mandate number of staff per shift and manage allocation of staff based on skill levels
Implementation of interventions – provided structure for AD to be managed
Organisational processes – workload/time allocation tasks hindered identification of acute deterioration
**Resource allocation and access to equipment**
Access to diagnostic equipment (bladder scanner, ECG)
Access to clinical assessment/observation equipment (vital sign monitor, thermometer, oxygen saturation probe, stethoscope)
Upskilling staff—programme to manage acute deterioration or access to relevant training to support the needs of a resident with acute deterioration
Access/relationships with GPs affiliated with care homes
Access to additional support services in the area
Lack of timely access/support from multi-disciplinary team/treatments. Care home staff felt they had to make decisions to transfer residents to hospital
Acute deterioration programmes come with opportunity cost. Care redirected to support the unwell resident(s). Inadequate resources to manage multiple unwell residents or cover routine care whilst staff are prioritising sick residents
High turnover of staff—requires regular ‘upskilling’ and training of staff
**Care home culture**
Staff’s relationships with residents are critical to identifying signs of acute deterioration. Developed over time, continuous care and frequent interactions
Resident and/or family requests influenced care decisions
Fear of litigation – care decisions being made through fear of legal repercussions as opposed to using clinical judgement/assessments to determine care
Low confidence – care home staff lacked confidence in managing acute deterioration Reinforced by feelings of working in isolation and external healthcare providers undervaluing their clinical assessments
Lack of autonomy to manage acute deterioration in residents – care staff felt they had little decision-making authority. Handed over ‘authority’ to external healthcare agencies
Hierarchies and role boundaries – existed between different staff groups. Overstretched staff were reluctant to report acute deterioration in residents as they felt it was not part of their role or through concerns it would increase their workload

The ratio of registered nurses to care home residents had a detrimental impact on early identification and management of acute deterioration. Studies reported RN to resident ratio ranging from 1:30 and 1:150. Two studies found that responses from healthcare professionals was delayed because of low RN to high resident ratios [29, 31]. RNs were unable to review the resident promptly due to competing responsibilities for other residents [30, 36–38].

Access to additional support and/or external healthcare providers was described in studies as variable, disjointed and dependent on local service configuration [30, 32, 34, 37–39]. The lack of timely multi-disciplinary support appeared to increase the likelihood of residents being admitted to hospital as RNs redirected their focus/care to arranging resident transfer to hospital rather than trying to manage the resident in situ [32, 34, 36, 38, 39].

The fear of litigation was a recurrent pattern identified in the majority of the included studies and directly influenced the management of acute deterioration including decisions to transfer residents to hospital [30–39]. This was particularly evident for RNs as they were reported as the ‘key individual’ who is accountable for managing any risks to residents. Additionally, nurses were reported as acting like brokers trying to satisfy ‘all sides’ including family, resident and doctor requests [30, 32, 34, 36–38]. There were instances reported where family members wanted their relative to receive treatment in hospital despite the nurse’s assessment establishing that this was unnecessary [30, 31, 38]. To help manage resident and relative expectations advanced care plans (ACPs) were described as a useful way to broach discussions around potential deterioration. However, four studies reported that ACPs or ‘emergency care plans’, were inadequate for acute deterioration scenarios and could further complicate decisions about whether to transfer to hospital [30, 32, 34, 37]. Partly, this was due to misunderstandings between ‘for active treatment’ and ‘not for resuscitation (CPR)’ and ACPs not being recognised as a legal document [34]. As a result, staff’s actions to escalate care e.g. calling for an ambulance was a means of managing any uncertainty surrounding the resident’s care and avoid potential litigation issues [30, 35, 36].

## Discussion

In this scoping review, we screened 399 journal articles and included *n* = 11 studies that featured qualitative data about how acute deterioration recognised and responded to in care home residents. Key findings are that most of the literature focusses on what happens when a resident deteriorates, without investigating how best to harness care home staff’s expertise in identifying deterioration. We found evidence of conflict between organisational policies and clinical judgement, issues around workforce including staffing ratios and skill-mix, and variability in the external support services offered to care homes to help manage acute deterioration and prevent hospital admissions. Finally, very little evidence of acute deterioration protocols or in-house procedures to manage this condition were identified.

The majority of sourced studies focused on resident transfer to hospital and not acute deterioration as the phenomenon of interest. Whilst there may be some overlap, in-depth and contextual nuances may have been missed that could provide valuable insights into how care homes manage this condition.

This literature review considers many contributing factors to hospital admission, including shared decision making, prognostication, advance care plans, the roles of multiple professionals and evaluations of hospital avoidance programmes [22, 40–42]. However, it does not consider the cascade of events that lead to care home staff identifying residents at risk of deterioration, or their decision to seek help external to the care home. A lack of data exists exploring how the use of care home observations and physiological observations inform subsequent shared decision-making about escalation and management of acute deterioration.

The review highlighted care home staff’s aspirations to manage the care of residents within the care home. However, due to a lack of adequate resources and infrastructure, hospital admissions were deemed unavoidable. Evidence demonstrates the risks and harms associated with hospital admission for older adults living with frailty, such as deconditioning, trauma, hospital acquired infections and poorer health outcomes [43–45]. Despite this, the only option for optimal treatment was hospital admission. In the UK, current services are not fit to support the care needs of older adults living in care homes [46]. This appears to be a global issue as highlighted by resource deficits in the articles (paucity of equipment, adequate training and timely access to sub-acute/acute care) [29, 31–33, 37, 40]. This requires greater consideration and restructuring of service provision to ensure the health and well-being of care home residents [46].

Within the UK, the uptake of EWS tools (NEWS/RESTORE) accelerated during the COVID-19 pandemic [47]. However, no evidence was identified detailing how they were being used in care homes and their impact on managing acute deterioration in residents with or without COVID. We still do not know the effect of these interventions, how they are being used to inform care, what is considered an appropriate response and whether they have been embedded into practice [18, 48]. Further research to determine the contextual factors surrounding the recognition and response to acute deterioration is needed, with particular attention to the processes of identification, how acute deterioration tools inform the management of this condition and what infrastructure is required to support care home staff when managing the care of acutely unwell residents.

## Strengths & limitations

The scoping review followed Joanna Briggs Institute scoping review methodology to ensure rigorous methods were adopted and minimised reviewer bias. A wide range of database searches were conducted to capture a broad range of data and ensure a comprehensive review. The study has provided an overview of the current available evidence regarding the topic of interest and identified the gaps in knowledge regarding acute deterioration in care homes.

Despite the broad search, the study mainly identified service evaluations and the implementation of acute deterioration tools/interventions in care homes that did not consider how care home workers identified or responded to acute deterioration. Further, the systematic development of the search strategy may have narrowed the number of articles retrieved due to the trade-off between specificity and sensitivity. It is likely that some care homes have in-house polices on the management of this condition that would not have been captured using the search strategy. The body of literature identified was small and the majority of studies conducted in Australia. Whilst there are likely organisational similarities in UK care homes, the findings from the review may not translate or apply as well to care home facilities in different countries. As per scoping review methodology, a critical appraisal of the quality of evidence was not conducted.

## Conclusion and implications

Recognising and responding to acute deterioration in care home residents is reliant on a complex and open system that encompasses multiple interrelated components. Currently, care home support services remain disjointed and vary across the country [49]. As the UK moves towards a systemised and integrated health and social care service and a focus on proactive, personalised care for older people in care homes [25], further intervention and evaluation studies are needed to enhance resident safety for this vulnerable group. Additionally, we need to fully understand the phenomenon of acute deterioration in the context of care homes and their residents and, to better outline what resources care homes need to manage this condition. This research should be conducted in different countries to understand how it is managed and to identify any differences/similarities in practice which may aid in the earlier identification and treatment of acutely deteriorating elderly patients.

## Supplementary Information

**Additional file 1: Appendix I.**

**Additional file 2: Appendix II.**

## Data Availability

The datasets used and/or analysed during the current study are available from the corresponding author on reasonable request.

## Abbreviations

EN: Enrolled Nurse
RN: Registered Nurse
PCA: Personal Care Assistant
GP: General Practitioner
RiR: Residential inReach Referral
RACF: Residential Aged Care Facility
NH: Nursing Home
CH: Care Home
NA: Nursing Assistant
