# Supplementary material for: Recognising and responding to acute deterioration in care home residents: a scoping review
Source: BMC Geriatr. 2023 Jun 29;23:399. doi: 10.1186/s12877-023-04082-y (PMC10308707; doi:10.1186/s12877-023-04082-y)
Supplement: Supplementary file 1 — Additional file 1: Appendix I. [file 12877_2023_4082_MOESM1_ESM.pdf]

**Modified data extraction form – taken from JBI scoping review methodology**<br>**Scoping review details**	
Scoping review title:	How do care home workers recognize and respond to acute deterioration in care home workers: a scoping review.
Review objective/s:	To identify published primary research as well as non-indexed and gray literature including policies, guidelines, and protocols regarding how Care Home staff recognize and respond to an acutely deteriorating resident.
Review question/s:	i) What is known from the existing literature of how care home staff recognize and respond to an acutely deteriorating resident?<br>ii) How do care home staff identify an acutely deteriorating resident?<br>iii) What do care home staff do in response to finding an acutely deteriorating resident?<br>iv) What mechanisms (if any) do care home staff use to help deliver care to an acutely deteriorating resident?
**Inclusion/exclusion criteria**	
Population	Care home workers providing direct care to care home residents. Unregistered and registered workers that deliver personal/health related care to residents.
Concept	AD refers to a severe, rapid illness that can be caused by infection, sepsis or a life threatening event such as an MI, cardiac arrest or CVA.<br>Studies that feature HOW care home workers recognize and respond to acute deterioration (AD) in care home residents. This can include any tools, systems or policies that care home workers use to aid the management of this condition. If care home workers use tools/interventions that have been designed by external sources, these can be included provided that they report on how they are being used by care home workers from the perspectives of care home staff participants.<br><br>What care home workers do – what actions do workers take once they recognise a resident with acute deterioration? This can also mean the decision not to act.
Context	Care homes refer to nursing and residential homes that provide 24/7 care and additional nursing care to residents. These are the permanent and long term addresses of care

	home residents. This review does not include care homes that offer respite care/holiday services.
Types of evidence source	Both qualitative and quantitative research will be considered alongside grey or non-indexed literature/social and health policies.
**Evidence source details and characteristics**	
Citation details (eg, author/s, date, title, journal, volume, issue, pages)	
Country	
Participants (details eg, age/sex and number)	
Context	
Methodology/methods/source of evidence	
**Details/results extracted from source of evidence (in relation to the concept of the scoping review)**	
What type of care home facility (residential, nursing or residential aged care facility?	
How do care home staff identify an acutely deteriorating resident? (e.g, through observations, monitoring signs and symptoms, “gut feeling”)	
What happened (response) as a result of identifying an acutely deteriorating resident (this includes the decision to not respond)?	
What mechanism did care home staff use to support recognition and response to acute deterioration (eg, tools/policies/guidelines/assessments or interventions)?	.
Barriers to recognizing and responding to acute deterioration in care homes.	
