# Supplementary material for: Recognising and responding to acute deterioration in care home residents: a scoping review
Source: BMC Geriatr. 2023 Jun 29;23:399. doi: 10.1186/s12877-023-04082-y (PMC10308707; doi:10.1186/s12877-023-04082-y)
Supplement: Supplementary file 2 — Additional file 2: Appendix II. [file 12877_2023_4082_MOESM2_ESM.docx]

MEDLINE OVID – full search strategy

| Search | Query | Results |
| --- | --- | --- |
| 1 | exp Home Nursing/ | 9398 |
| 2 | exp residential facilities/ or assisted living facilities/ or homes for the aged/ | 52974 |
| 3 | ("nursing home*" or "residential home" or "age? care facilit*" or "care facilit*").ti,ab. | 51785 |
| 4 | ("RACF" or "residential aged care facilit*").ti,ab. | 733 |
| 5 | 1 or 2 or 3 or 4 | 89998 |
| 6 | exp Clinical Deterioration/ | 304 |
| 7 | (("rapid*" or "acute*") adj2 unwell).ti,ab. | 346 |
| 8 | exp Acute Disease/ | 213558 |
| 9 | deterior*.mp. | 129458 |
| 10 | ("acute* unwell" or "rapid* unwell" or "acute* deterior*" or "rapid* deterior*").mp. | 4260 |
| 11 | (("recognis*" or "identif*" or "respon*" or "manag*") adj3 "deterior*").mp. | 2096 |
| 12 | 6 or 7 or 8 or 9 or 10 | 341343 |
| 13 | 5 and 11 and 12 | 30 |
| 14 | Limit 13 to (English language and yr=”2009-current” and “all aged (65 and over)”) | 10 |
